# When Methotrexate Is Not Enough: Hysteroscopic Management of an Angular Pregnancy With Discordant Beta-Human Chorionic Gonadotropin (b-hCG) and Ultrasound Findings

**DOI:** 10.7759/cureus.107054

**Published:** 2026-04-14

**Authors:** Francesco Pio Toscano, Virginia Gargano, Lucio Cipullo, Roberta Gallo, Raffaella Del Papato, Giuseppe Laurelli

**Affiliations:** 1 Public Health, Università degli Studi di Napoli Federico II, Napoli, ITA; 2 Obstetrics and Gynecology, Azienda Ospedaliera Universitaria San Giovanni di Dio Ruggi d’Aragona Scuola Medica Salernitana, Salerno, ITA

**Keywords:** angular pregnancy, b-hcg monitoring, hysteroscopy, methotrexate, mini-resectoscope

## Abstract

An angular pregnancy is a rare form of ectopic implantation. We report a 30-year-old primigravida presenting with an abnormal beta-human chorionic gonadotropin (b-hCG) trend. At presentation, her b-hCG was 1,950 mIU/mL. Ultrasound showed an angular gestational sac with increased vascularization and deep myometrial extension. Methotrexate was administered, achieving biochemical response but with persistent suspicious ultrasound findings, suggesting ongoing trophoblastic activity. Due to this discordance, hysteroscopic management was performed. The gestational tissue was exposed and removed using a bipolar mini-resectoscope under direct visualization. The patient was discharged the same day. Hysteroscopy may represent a safe and effective option in selected cases with discordant biochemical and imaging findings.

## Introduction

Ectopic pregnancy is the implantation of the gestational sac outside the uterine cavity. Ectopic pregnancies can be classified into extrauterine and intrauterine. Tubal pregnancy represents the most common type, accounting for approximately 95% of all ectopic pregnancies [1]. Intrauterine ectopic pregnancies, including angular, interstitial, cervical, and cesarean section scar pregnancy, are considerably less frequent and represent a minority of cases [1].

A ruptured interstitial, angular, or cornual ectopic pregnancy represents a surgical emergency due to the risk of severe hemorrhage; therefore, early diagnosis and prompt management are essential [2,3]. Systemic methotrexate is considered the first-line treatment for selected cases of ectopic pregnancy [4], but an angular pregnancy may also be amenable to a less invasive surgical approach, such as hysteroscopic resection [2]. This may be a minimally invasive alternative approach that allows the gestational sac to be removed while preserving the anatomy and, therefore, the fertility of the patient.

The terminology surrounding angular, cornual, and interstitial pregnancies remains inconsistent in the literature, with these entities often used interchangeably [5], despite representing distinct anatomical and clinical conditions. An angular pregnancy is an uncommon obstetric condition, first reported by Kelly in 1898 [6], defined as the implantation of the embryo within the endometrial cavity at the superolateral angle of the uterine cavity, medial to the utero-tubal junction. Given its anatomical proximity to this junction, angular pregnancy is frequently misidentified as either an interstitial or a cornual pregnancy. The term “cornual pregnancy” refers specifically to an intrauterine implantation occurring within a structurally abnormal uterus, such as a unicornuate, bicornuate, or septate variant, whereas a peripheral trophoblastic implantation arising in a morphologically normal uterus is more appropriately classified as either “interstitial” or “angular” pregnancy [7]. 

This distinction is clinically crucial, as interstitial pregnancies are associated with a significantly higher risk of rupture and life-threatening hemorrhage [3]. These pregnancies are often misdiagnosed and associated with subsequent complications because of their deep implantation, which may delay detection and permit further growth, thereby increasing the risk of rupture and severe hemorrhage. However, differentiating these entities in early pregnancy remains challenging, even with high-resolution transvaginal ultrasound [3]. Management remains controversial, particularly when biochemical and imaging findings are discordant.

This article reports a case of a viable angular pregnancy successfully treated with bipolar mini-resectoscopy after failed systemic methotrexate administration. It defines the hysteroscopic treatment step-by-step, highlighting a possible role of hysteroscopy in such cases.

## Case presentation

A 30-year-old woman, primigravida, was referred to our unit due to an abnormal serum human chorionic gonadotrophin (b-hCG) trend and vaginal spotting. On admission, her serum b-hCG level was 1,950 mIU/mL. Her vital signs were stable. She reported mild vaginal spotting, and her hemoglobin concentration was 10.5 g/dL (reference range: 12-15 g/dL).

**Figure 1:**
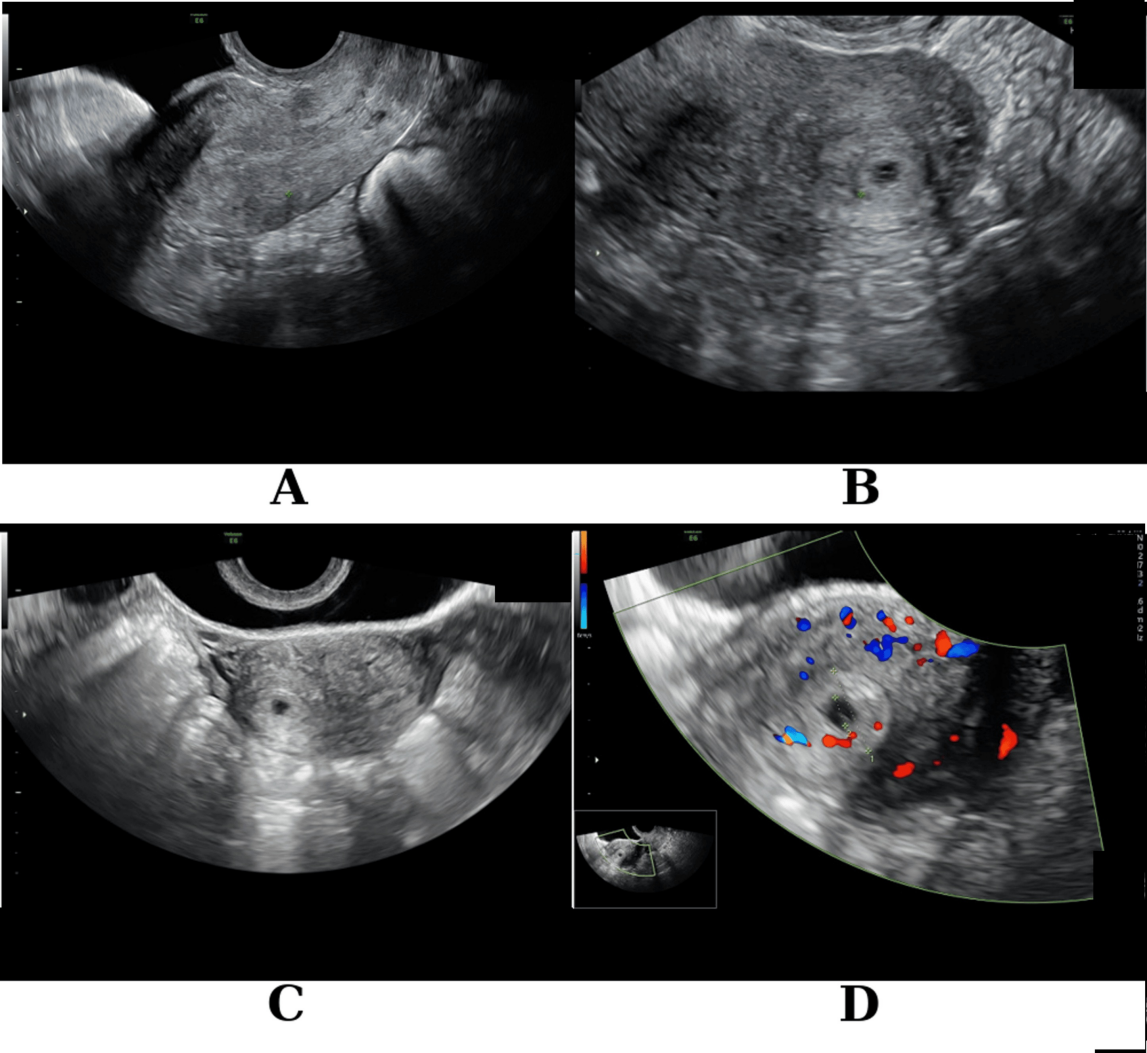
Transvaginal ultrasound findings of angular pregnancy with myometrial extension (A) Empty endometrial cavity; (B) Eccentrically located gestational sac; (C) Apparent intramyometrial localization of the gestational sac, mimicking an interstitial implantation; (D) Color Doppler imaging demonstrating persistent peripheral vascularization of the gestational sac following systemic methotrexate administration.

Transvaginal ultrasound examination (Figure 1) demonstrated an angular gestational sac implanted at the posterolateral angle of the right uterine cavity, medial to the utero-tubal junction, with increased peripheral vascularization on color Doppler assessment, and evidence of deep myometrial extension toward the uterotubal junction.

Three-dimensional sonographic reconstruction confirmed a T-shaped uterine cavity (U1a as per the European Society of Human Reproduction and Embryology/European Society for Gynaecological Endoscopy (ESHRE/ESGE) classification [8]), characterized by thickened lateral walls and a restricted fundal region, which is thought to have predisposed to the anomalous implantation site.

The initial serum b-hCG concentration at admission was 1,950 mIU/mL. Serial b-hCG measurements performed prior to admission demonstrated an abnormal and fluctuating trend (Table 1), characterized by an initial decline followed by a plateau and subsequent increase.

**Table 1:** Serial b-hCG measurements before and after admission Day 0 corresponds to the day of hospital admission. Negative values (−) indicate days prior to admission, whereas positive values (+) indicate days following admission; b-hCG: beta-human chorionic gonadotropin.

Day	Event	b-hCG (mIU/mL)
-17	Pre-admission	2537
-16	Pre-admission	2149
-13	Pre-admission	1107
-10	Pre-admission	653
-7	Pre-admission	640
-4	Pre-admission	789
0	Admission	1950
+1	-	2036
+3	-	2350
+5	Methotrexate (MTX) administered	2753
+7	Post-MTX	3130
+8	Post-MTX	3356
+9	Post-MTX	3426
+12	Post-MTX	2729
+13	Post-MTX	2249
+14	Hysteroscopic treatment	-
+19	Follow-up	80.3

This pattern was not consistent with a normally progressing intrauterine pregnancy and suggested early trophoblastic dysfunction, raising suspicion of an ectopic or abnormally implanted pregnancy.

An initial expectant management was adopted. However, b-hCG levels did not decrease and instead showed a progressive increase on follow-up evaluations. The patient was therefore counselled again regarding the risks associated with the progression of this type of pregnancy. She was informed of the high risks of uterine rupture, severe maternal hemorrhage, and the potential need for emergency hysterectomy associated with this abnormal implantation [9]. Given the location of the gestational sac at the uterine angle, with extension toward the uterotubal junction, these risks were considered particularly relevant. Accordingly, the patient opted for termination of the pregnancy with the aim of preserving fertility. The available management options were discussed with her. She chose medical treatment with methotrexate, administered as a single intramuscular dose calculated according to body surface area (50 mg/m²) [4]. 

Despite an adequate biochemical response, with serum b-hCG levels showing a reduction of more than 15% seven days after methotrexate administration (Table 1), ultrasound findings remained concerning, demonstrating increased perilesional vascularization and persistence of an evolving gestation (Figure 1D). These findings suggested ongoing trophoblastic activity in a high-risk implantation site, prompting surgical management. Therefore, a minimally invasive hysteroscopic approach was selected.

The procedure was performed in the operating room under deep sedation. A vaginoscopic diagnostic hysteroscopy was initially carried out using a 5-mm continuous-flow operative hysteroscope (Bettocchi hysteroscope, 15 Fr; Karl Storz, Tuttlingen, Germany) with normal saline solution for uterine distension at low pressure. This revealed a small uterine cavity with irregular morphology, consistent with a slightly hypoplastic T-shaped uterus (U1a, ESHRE/ESGE classification [8]). 

**Figure 2:**
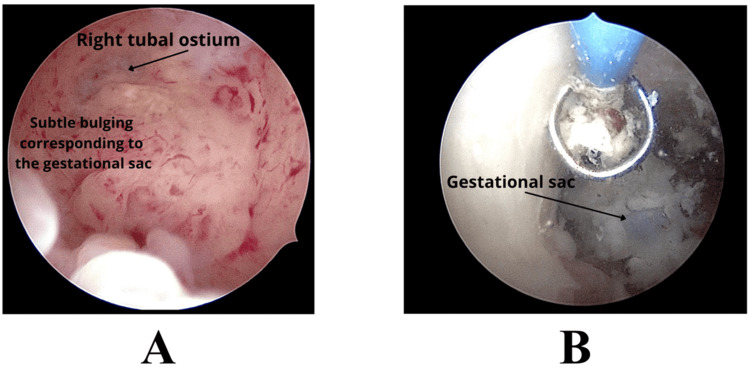
Hysteroscopic visualization and enucleation of an angular pregnancy (A) Diagnostic hysteroscopy using a Bettocchi hysteroscope showing a subtle bulging at the right uterine angle, close to the tubal ostium, suggestive of the underlying gestational sac; (B) Hysteroscopic view after progressive resection, showing the gestational sac following mobilization and enucleation.

On hysteroscopic evaluation (Figure 2A), a clearly defined gestational sac was not immediately visible. Instead, a localized bulging and highly vascularized area at the right uterine angle was observed, corresponding to the site of implantation.

In consideration of these findings, the procedure was continued using a mini-resectoscope with bipolar energy. An initial superficial slicing of the overlying tissue was performed using a bipolar loop in order to expose the gestational sac. Subsequently, “gentle curettage” with the loop electrode was carried out to progressively mobilize the gestational tissue from its implantation site (Figure 2B). Once adequately mobilized, the gestational sac was completely removed en bloc under direct hysteroscopic vision. Final inspection confirmed regular hemostasis. Intraoperative ultrasound control was performed. No intraoperative complications were observed. 

However, even minimally invasive surgical approaches may still carry a risk of significant bleeding, and patients should always be counselled about the possible need for emergency hysterectomy [2,9,10]. In our case, the procedure was performed in an operating room setting, with full readiness for immediate conversion to laparoscopy or emergency hysterectomy if required. 

**Figure 3:**
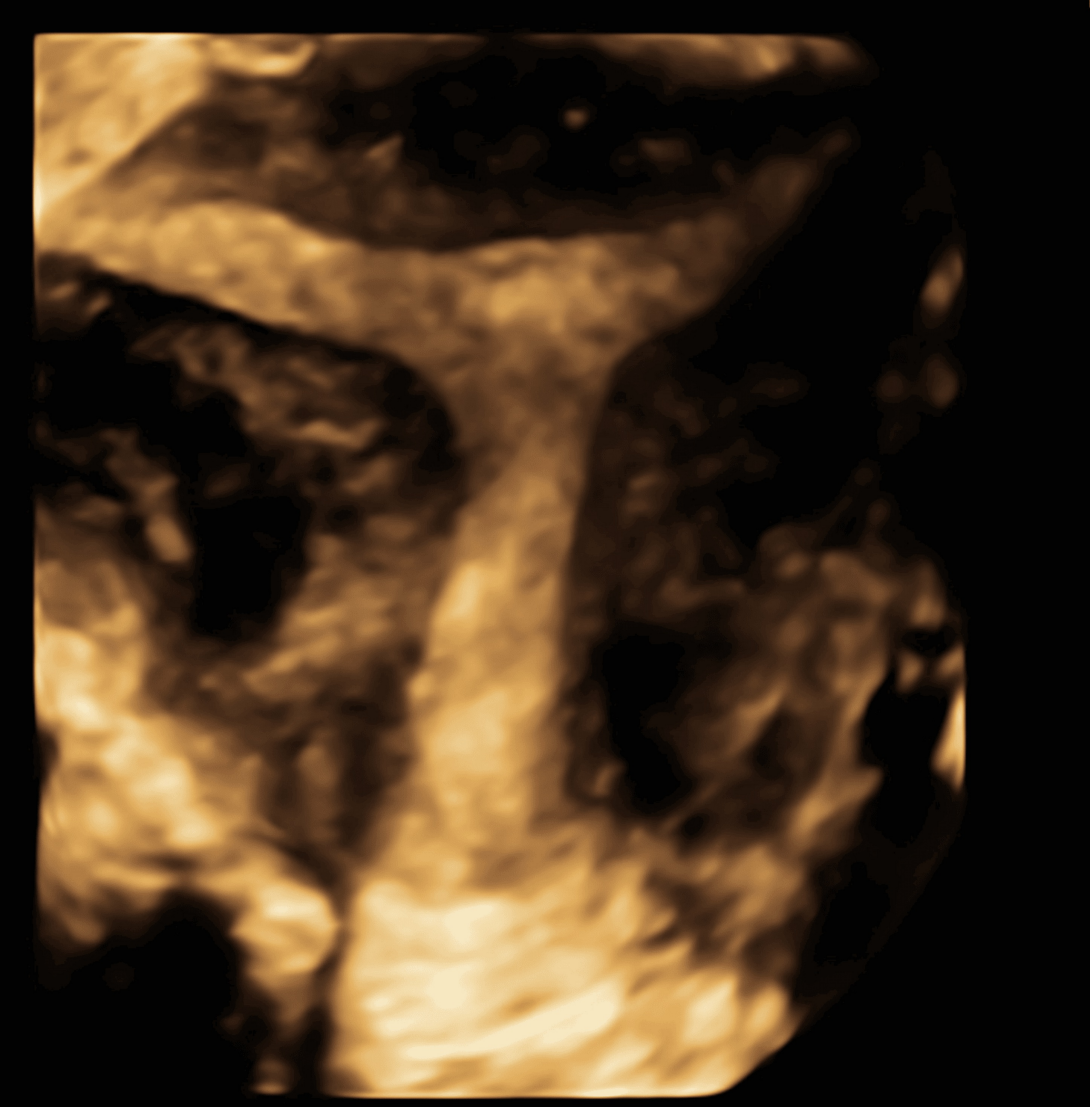
Follow-up 3D ultrasound

The patient was discharged on the same day of the procedure after approximately 12 hours of observation. At the five-day follow-up, her serum b-hCG levels showed a marked decline to 80.3 mIU/mL, and the transvaginal ultrasound (Figure 3) confirmed complete resolution of the pregnancy with no residual gestational tissue.

b-hCG levels subsequently declined to negative values, further confirming treatment success.

## Discussion

Methotrexate treatment may be unsuccessful in a subset of patients, requiring subsequent surgical management [9]. A biochemical response to methotrexate does not guarantee resolution of trophoblastic activity. In the present case, despite a decline in b-hCG levels, ultrasound findings remained suspicious for persistent active implantation, highlighting a clinically relevant discordance between biochemical and imaging parameters.

This scenario may lead to an underestimation of the ongoing disease if management is based on biochemical trends alone. In addition, the anatomical location of implantation at the uterine angle, with extension toward the uterotubal junction, may further increase the risk of severe complications such as uterine rupture and hemorrhage [9].

The differentiation between pregnancies located near the uterotubal junction remains complex, and inconsistent terminology has contributed to diagnostic ambiguity. The interchangeable use of the terms angular, cornual, and interstitial pregnancy may lead to misclassification, with intrauterine pregnancies being incorrectly managed as ectopic pregnancies [6]. In this context, the presence of surrounding endometrium has been proposed as a reliable sonographic sign to identify eccentric intrauterine implantation and reduce the risk of overtreatment [3]. However, in the present case, this reassuring feature was not clearly identifiable. Instead, ultrasound demonstrated deep myometrial extension and increased perilesional vascularization, raising concern for a more invasive implantation pattern. The abnormal and fluctuating b-hCG trend was not consistent with a normally progressing intrauterine pregnancy, further supporting the need for active management.

Moreover, recent evidence suggests that many pregnancies previously classified as angular may follow a benign course and can be successfully managed, with high live birth rates and low complication rates [5]. However, this approach may not be appropriate in the presence of discordant biochemical and imaging findings or signs of invasive implantation, as observed in the present case.

In such cases, hysteroscopy allows direct visualization of the implantation site and targeted removal of trophoblastic tissue under direct vision. Compared to blind or indirect approaches, this technique enables more precise and controlled tissue removal. Previous reports have shown that hysteroscopic management of abnormal implantations can be safe and effective, with high success rates and low complication rates [2,10].

The rapid decline in b-hCG levels to 80.3 mIU/mL within five days and same-day discharge suggest that there are potential clinical and logistical advantages to this approach.

## Conclusions

In experienced hands, hysteroscopy represents a valuable minimally invasive option in selected cases of abnormal implantation. When medical management is insufficient, it allows precise, fertility-preserving treatment under direct visualization. A biochemical response to methotrexate does not necessarily indicate complete resolution, and discordance with ultrasound findings should prompt further evaluation.

Timely hysteroscopic management may achieve definitive treatment while minimizing potential complications. Although the rarity of this condition precludes definitive conclusions, hysteroscopy may be considered in selected cases, particularly in the presence of discordant biochemical and ultrasound findings or anatomical variants predisposing to abnormal implantation.
